# Review of the Problems of Additive Manufacturing of Nanostructured High-Energy Materials

**DOI:** 10.3390/ma14237394

**Published:** 2021-12-02

**Authors:** Olga Kudryashova, Marat Lerner, Alexander Vorozhtsov, Sergei Sokolov, Vladimir Promakhov

**Affiliations:** 1 Laboratory for High Energy and Special Materials, National Research Tomsk State University, Lenin Avenue, 36, 634050 Tomsk, Russia; olgakudr@inbox.ru (O.K.); lerner@ispms.tsc.ru (M.L.); abv1953@mail.ru (A.V.); sokolovsd95@gmail.com (S.S.); 2 Institute for Problems of Chemical and Energy Technologies, Siberian Branch of the Russian Academy of Sciences, St. Socialist, 1, 659322 Biysk, Russia; 3 Institute of Strength Physics and Materials Science, Siberian Branch of the Russian Academy of Sciences, Ave. Academic, 2/4, 634055 Tomsk, Russia

**Keywords:** additive manufacturing, thermite, high-energy materials, 3D printing

## Abstract

This article dwells upon the additive manufacturing of high-energy materials (HEM) with regards to the problems of this technology’s development. This work is aimed at identifying and describing the main problems currently arising in the use of AM for nanostructured high-energy materials and gives an idea of the valuable opportunities that it provides in the hope of promoting further development in this area. Original approaches are proposed for solving one of the main problems in the production of nanostructured HEM—safety and viscosity reduction of the polymer-nanopowder system. Studies have shown an almost complete degree of deagglomeration of microencapsulated aluminum powders. Such powders have the potential to create new systems for safe 3D printing using high-energy materials.

## 1. Introduction

In recent years, there has been explosive growth in interest toward additive manufacturing in many scientific research areas. This can be seen from the increase in the number of publications on this topic [1], as well as the respective economic indicators; the current additive manufacturing (AM) market size has already exceeded $1300 million. In practice, AM saves time and costs from the design stage to production [2].

Additive manufacturing technologies have been developing for over 30 years. While at the onset, they consisted of relatively simple 3D printing of polymer prototypes, now it is possible to use AM to obtain metallic or non-metallic prototypes or functional products that do not require mechanical post-processing. One of the examples is additive manufacturing by means of layer-by-layer laser fusion of powder [3,4,5].

The main direction of AM application today is the production of functional products for aerospace and automotive [6] industries and biomedicine, as well as manufacturing of electronic devices and measuring equipment, etc. [1].

Despite significant advances in AM in recent years, some areas remain problematic. These include AM of high-energy materials (HEM).

Recently, interest has arisen in high-energy nanocomposites. Those make it possible to create miniature energy systems based on nanomaterials with more powerful oxidation kinetics than macroscale materials. Such composites are called “reactive materials” [7,8], “metastable intermolecular composites” [9,10], or “pyrolants” [11,12]. They are prominent in terms of high heat release per unit mass of substance. Furthermore, being nanoscale, they have the potential for precise targeting applications. However, their manufacturing technologies should also differ from conventional ones.

Conventional technologies such as pressing or casting are used for manufacturing products from high-energy materials. These technologies also suffer from imperfections: resulting products may contain pores and cracks [13], and some technological stages are unsafe.

Additive manufacturing technologies are the most promising field for the fabrication of products from high-energy nanocomposites since they are commonly and widely used for the creation of nanocomposite products [1,14]. Additive manufacturing covers a wide range of technologies for the layer-by-layer deposition and synthesis of objects based on computer-designed 3D models. The advantage of such technologies lies in the creation of objects of complex geometry with high resolution, which is important for the use of nanomaterials. However, not all technologies in the AM toolbox are suitable for handling high-energy materials.

Methods for creating planar (2D) structures from reactive materials are outside the scope of this review (unless they are a variation of 3D), and they are considered in detail in [14].

In this research, we rely on the results of the review [14] and earlier work [15] as well as on new articles that have been published later. We focus on the problems of the additive manufacturing of high-energy nanocomposites and the possible ways of solving them.

## 2. High-Energy Composite Additive Manufacturing Technologies

### 2.1. Material Extrusion

Material extrusion is the first and, currently, the most popular additive manufacturing process for a wide range of products in terms of affordability and reliability. Material extrusion is a 3D inkjet printing process whereby the material is fed through a nozzle or jet. This principle of operation is mostly used in inexpensive desktop 3D printers. The requirement for materials for the fabrication of items is the ability to push the material through the nozzle. Any pasty material (sometimes preheated) can be used for “drawing” 2D cross-sections of a certain 3D model.

**Figure 1:**
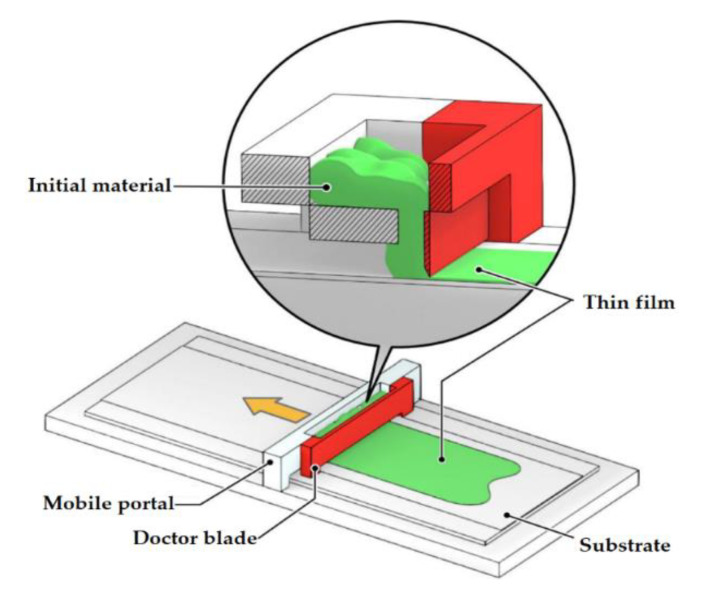
Material extrusion. Fabrication of a thin HEM film using the Doctor Blade Casting method.

A variation of the process under consideration is the creation of flat structures, for example, screen printing for pyrotechnic applications [16] and Doctor Blade Casting [17]. The Doctor Blade method can operate at speeds of up to several meters per minute and is suitable for coating substrates with a very wide range of wet film thicknesses ranging from 20 microns to several hundred microns. Here, an HEM suspension with a dissolved binder is applied to the substrate using a knife with a specific gap (Figure 1). A team from Texas Tech University led by Professor Pantoya has published a series of articles on the fabrication of thermite-based films using this approach [18,19,20].

**Figure 2:**
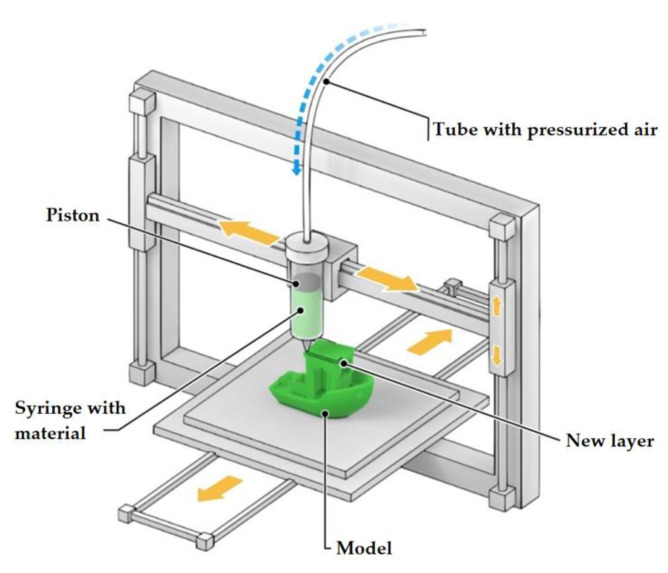
Direct printing method, a schematic diagram.

The extrusion process is also used for the formation of more complex 3D structures. To this end, the “ink” is applied layer by layer through the hole in the 3D printer head (Figure 2). The respective 3D printing method is called DIW (Direct Ink Writing). Initially, the liquid ink solidifies on the substrate as a result of solvent evaporation or binder deposition, thus creating a volumetric product structure. The minimum size of printed structures can be less than 1 micron, depending on the size of the nozzle and the physicochemical properties of the ink. Thanks to its relative simplicity, DIW is widely used for the fabrication of HEM products [21,22,23,24,25,26].

The pen-type technique is a variation of this method. Here, a specially prepared ink containing a dispersed phase (a nano- or micro-sized aluminum powder, for example) and a binder are used. The ink fills the syringe, and the extrusion process is actuated by pressurized air. The adjustment of air pressure allows for controlling the printing speed and setting up the technology in accordance with the ink viscosity. However, manufacturing articles with a solid content of over 90%, using the pen-type technique, is still a problem. This is because high pressure is required, which also results in the slurry stratification phenomenon, i.e., the separation of the binder from the solids [27].

To overcome the limitations associated with high ink viscosity, an ultrasonic technique with high frequency and amplitude oscillations is used [28] (up to 30 kHz and 20 µm, respectively). However, ultrasound creates flow hysteresis problems leading to inaccuracies in geometry, nozzle erosion and ink degradation due to high stress.

The main problem of using this technology for the fabrication of products from high-energy compositions is their hazardous nature. Reactive materials are susceptible to ignition, deflagration, detonation or explosion when subjected to shear stress and friction during extrusion as they travel through valve and nozzle assemblies. In addition, it is necessary to meet ink viscosity requirements to create the conditions for the extrusion itself (i.e., material passage through the nozzle) [14,29]. There are significant difficulties to be overcome, especially when using a suspension with a very high particle content (>80 vol.% of particles). Work [29] is devoted to the investigation of a polymer binder used in high-energy compositions from the point of view of the viscosity of the suspension and its suitability for printing.

In general, DIW direct printing technology achieves a relatively high slurry-solid content and appropriate viscosity of the material using special techniques (ultrasound) and careful ink formulation designs, individually designed for each application.

One of the important advantages of this process, as applied to high-energy materials, is the relatively low operating temperatures, which reduce the probability of ignition. The operating temperatures are determined by the physical and chemical characteristics of the material.

### 2.2. Fused Deposition Modeling (FDM)

**Figure 3:**
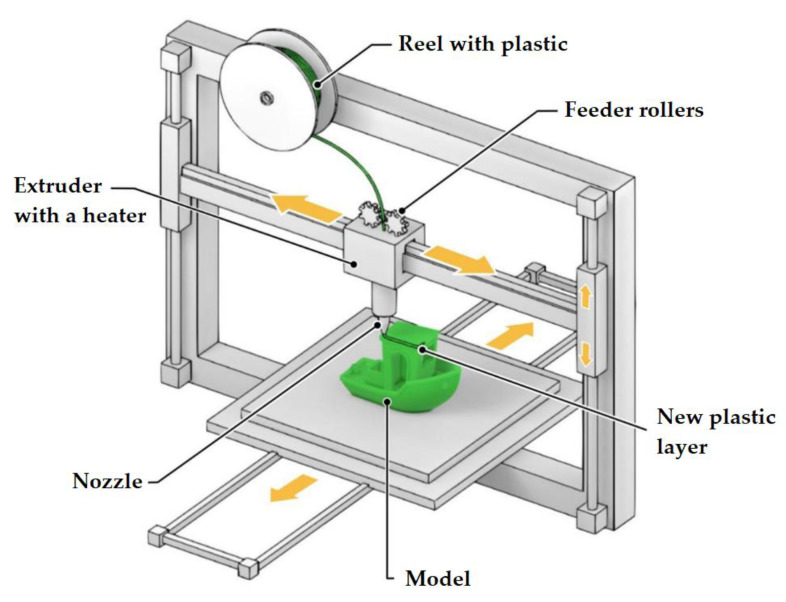
Modeling by layer-by-layer deposition, a schematic diagram.

FDM is a widely used additive manufacturing technology. It is based on the use of a plastic filament which is deposited in layers through a heated extruder onto a substrate (Figure 3). Typical spatial resolutions achieved by the FDM method lie in the range of 100–500 µm.

Today, the FDM printing process has matured to an industrial level, and inexpensive commercial printers are used in the manufacturing process. However, there is little data on the fabrication of high-energy composites based on this technology. The reason for this is obvious: FDM involves a rather high degree of material heating to increase its fluidity. This limits the range of materials that can be printed with FDM to those with a melting point significantly lower than the reaction temperature [30,31].

Another approach that still does not completely eliminate ignition during printing consists of filling a thermoplastic polymer filament with solid HEM particles [32].

The main difference between FDM and DIW is the required feedstock viscosity (for example, 0, 1–103 Pa·s [33] for DIW and >103 Pa·s for FDM). The application of FDM to high-energy materials requires the incorporation of high concentrations of reactive particles into the polymer filament to maintain high reaction rates. However, from the point of view of uniform distribution of the components and reduction of material viscosity during printing, lower particle concentrations are recommended [34].

Most of the recent works devoted to the use of FDM for the creation of HEM products are focused on the use of poly (vinylidene fluoride) (PVDF), which can serve both as a filament backbone in printing and as an oxidizing agent during the reaction [34,35,36,37]. In particular, nano-aluminum (n-Al) and polyvinylidene fluoride (PVDF) are attractive fuel and oxidizing materials due to the high energy density of n-Al as well as high oxidation potential and excellent mechanical properties offered by PVDF [38].

Summarizing the research findings obtained on the use of the FDM method for the additive manufacturing of HEM, it can be assumed that this technology has high potential. The main disadvantages of FDM are the limited surface finishing capabilities and precision as well as poor mechanical properties of the products [39]. Balancing between the material viscosity and its reactivity also remains a problem as the relatively high temperature of the filament compromises safety during printing.

### 2.3. Photopolymerization (Laser Stereolithography (SLA), Digital Light Processing (DPL))

**Figure 4:**
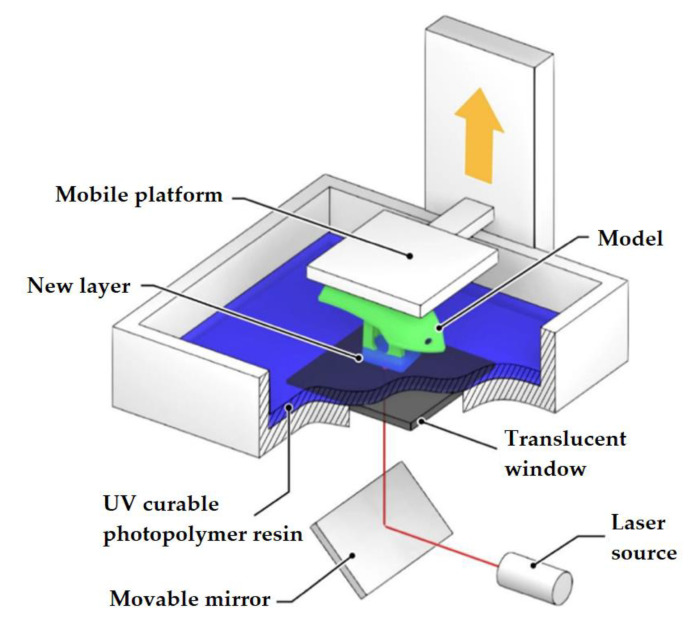
SLA method.

**Figure 5:**
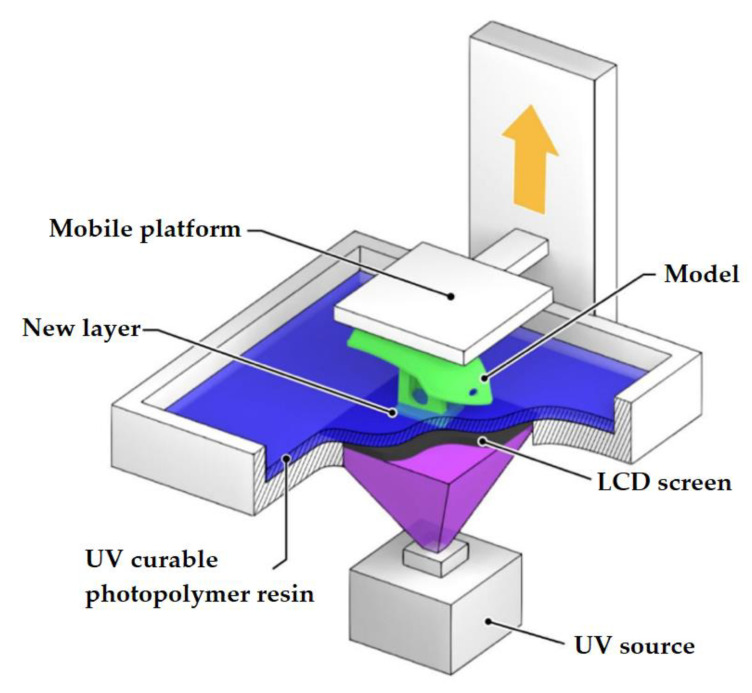
DLP method.

Vat Photopolymerization is a 3D printing process that implements a light source to cure liquid photocurable resins (photopolymers). The key difference between the two technologies lies in the type of light source used for curing the material: in SLA (Figure 4), it is ultraviolet light, and in DLP (Figure 5), it is visible light. Normally, the light is projected downwards on the printed material in SLA systems, and from under the printed material (through a transparent surface), in DLP systems.

A 3D printer involved in the implementation of vat photopolymerization is equipped with a container with photopolymer resin, which is cured by the light source. SLA printers use a vat with liquid photopolymer resin that is cured by UV radiation. Laser beam processing is done layer by layer. After one layer has been treated, the printer platform is shifted by a distance equal to the layer thickness (0.05–0.15 mm). The resin-filled plate then passes over the cross-section of the item and re-coats it with new material. The next layer is formed on this liquid surface and is joined with the previous one. Thus, an entire 3-dimensional object is formed.

DLP uses visible light projectors such as arc lamps. In this case, each layer of the object being fabricated is projected into a vat of liquid resin, which hardens layer by layer as the platform is raised or lowered. Thanks to this treatment method, DLP is faster than most 3D printing methods.

There are a number of works devoted to SLA application in energy product manufacturing: fuel pellets for hybrid engines [40], gun powders [30,41], compositions based on ammonium perchlorate [42] and cannon fuels [43]. DLP for energetic materials was used in [44,45]. Some works reported the use of a combination of DIW and SLA: the composite is first deposited by extrusion and then cured by UV radiation [46,47].

The use of photopolymerization processes in a vat imposes a number of requirements for the materials [45]:

- All the components must be chemically compatible.
- The viscosity of the material should be sufficiently low, preferably below 20 Pa⋅s [48].
- The material must be sufficiently stable over time, i.e., sedimentation must not occur for several hours.
- The material must be sensitive to the wavelength range of the light source used.
- The material must be promptly cured, preferably within a few seconds.
- Light penetration into the material (including light scattering) must be limited to provide sufficient resolution in all directions.
- Once illuminated, the material must have sufficient mechanical strength to withstand transportation, treatment and use.

Additional requirements largely depend on the intended use of the printed product. In the case of energy cells, it can be said that the following requirements must be met:

- The energy resin must not decompose when exposed to the light source used for curing.
- The energy properties (e.g., heat of explosion, combustion rate, detonation velocity) must be sufficient for the intended use.

The main advantages of the photopolymerization methods are high resolution and high performance. The light-induced process eliminates the use of toxic hardeners. The main disadvantage is the need to use polymers that are curable by UV or visible light. This significantly limits the list of materials available for printing.

### 2.4. Powder Printing (Binder Jetting, Powder Bed Printing)

**Figure 6:**
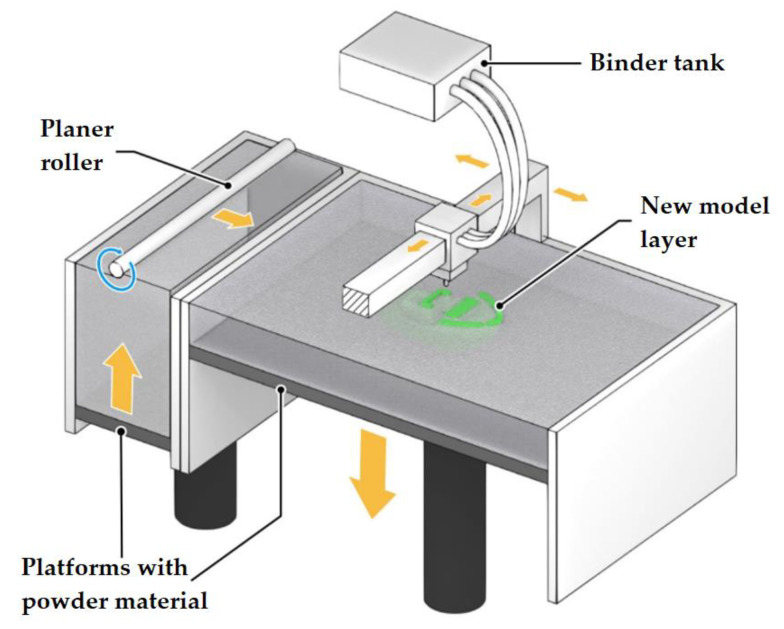
Binder Jetting (Powder Bed Printing), a schematic diagram.

Two materials are used in printing with this technology: a base material in the form of a powder and a liquid binder. The binder is sprayed by the printer head, and the powder particles stick together to form a solid 3D model (Figure 6). The powder is poured into the first chamber and fed into the second chamber by rolling the required amount of powder with a roller. The powder is poured onto the construction platform lowered to a depth equal to the first layer height. A liquid adhesive binder is fed through the inkjet printer head. After laying one layer, the platform is lowered to a depth equal to the height of the next layer. Then the powder is rolled out of the first chamber into the construction chamber. All subsequent layers are deposited in the same way. After completing the last layer, the 3D model is extracted, and the excess powder is peeled off. Initially, this technology was used to create plaster molds and casting molds.

The difficulty of using this technology with high-energy materials lies primarily in the fact that post-processing is required to eliminate mechanical defects and increase strength [49]. Apart from that, the removal of excess reactive powder can also be challenging [14].

### 2.5. Powder Sintering Technology (Selective Laser Sintering, SLS)

There are other additive manufacturing technologies that are less suitable for handling reactive materials (most often, for safety considerations). However, literary sources describe attempts to use them.

**Figure 7:**
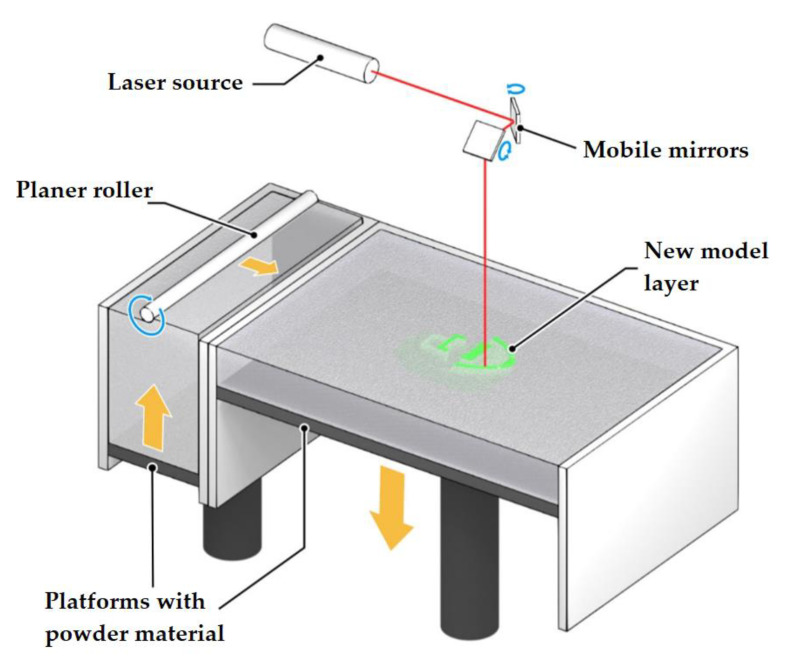
Selective laser sintering (SLS), a schematic diagram.

SLS or Selective Laser Sintering is an additive manufacturing technology based on layer-by-layer sintering of powder materials using a laser beam [50]. The laser beam selectively activates powder particles, causing them to partially melt and coalesce with neighboring particles, thus forming a monolithic layer. The SLS technology provides only partial melting of the surface of the particles. This is necessary for sintering them together. However, the temperature in the laser treatment area is close to the melting temperature of the powder. The process of manufacturing a product using the SLS technology has the following stages (Figure 7): heating the powder to a temperature close to the melting point; feeding the powder to the fabrication chamber; sintering the powder in the required areas with a laser beam; feeding the next layer of powder (the fabrication chamber goes down one layer), etc.

The advantages of the SLS technology are high fabrication precision for products of complex geometric shapes, high speed and performance, excellent mechanical properties of products and the absence of wastes. The disadvantages include the high cost of equipment.

The research work [51] investigated the possibility of using the SLS technology for handling highly explosive composites such as RDX and TNT. The idea of modifying the method was that explosive particles are covered with a polymer shell with a low melting point. This will ensure easy sintering of particles among themselves while avoiding the initiation of explosives. The SLS concept has been successfully demonstrated on explosive simulators coated with polycaprolactone (PCL). However, the authors still recommend using technologies for the additive manufacturing of explosive materials without the use of intense laser energy (for example, powder printing).

## 3. Reactive Materials for Printing, Problems of Their Preparation and Use

The last two decades have seen remarkable developments in the field of energetic materials. There has been a gradual transition from the use of nitrocarbon energetic materials, such as TNT, RDX and CL-20, to microstructural composites and nanothermites [52]. Thermite is a powder mixture composed of oxygen donor particles, such as a metal oxide and an oxygen scavenger represented by a reducing metal (fuel). Nanothermites are produced from finely dispersed powders where particle sizes normally range between 1 and 100 nm. Aluminum nanopowder has proven to be one of the best fuels for nanothermites. The commercial availability of stable aluminum nanopowders with narrow particle size distributions and well-defined metal contents has stimulated research in this area [53].

In contrast to micro-sized energetic materials, nanothermites have a higher specific surface area (~10–50 m^2^/g), a lower ignition temperature, a higher energy density (up to 50 MJ/kg), a shorter ignition time, a higher burning rate [54,55] and a lower impact sensitivity (<4–35 J) [52]. The combustion of nanothermites often occurs due to the dispersion of condensed products by gases (advection), which provides a flame propagation velocity of up to 2400–2600 m/s [56,57,58]; this is significantly higher than that of micron-sized thermites.

In particular, such exceptional properties of nanoenergy composites (i.e., thermites, combinations of metal-fuel/metal oxide particles) find their applications in ammunition, pyrotechnics and microcircuits of microelectromechanical systems (MEMS). Very high reaction rates (often in the form of detonation) are useful in the above applications. However, due to the high sensitivity, there is a certain hazard during their production and handling.

In addition to high sensitivity, there are other factors limiting the use of nanothermites [14], including stability problems, for example, oxidation [59] or coalescence [60,61,62] during storage; the difficulty of obtaining a homogeneous composite mixture [55,63]; gradual increase in the viscosity of compositions with a binder [64]. Furthermore, toxicological hazard remains an important issue when handling nanopowders [52,53].

In addition to the reactive material, energetic inks contain a binder. The main role of the binder is to improve the mechanical properties of the composite [65]. The binder encapsulates the powder particles, reducing their oxidation and coalescence [62]. Another possible role for the polymeric binder lies in increasing the energy of the composition if the binder reacts with the filler particles. For example, fluoropolymers oxidize aluminum [36,66,67,68], and 80% more energy is generated in the process as compared to the formation of aluminum oxide [11].

An integral part of the ink used in most additive manufacturing technologies is a solvent or dispersion medium. The use of liquid media increases safety by reducing the sensitivity to electrostatic discharge as compared to the use of dry powders [69].

The suspension used in additive manufacturing (e.g., DIW) must be properly designed to meet the following requirements: uniform distribution of particles, fast solidification kinetics and suitable rheology for substance flow and shape retention [29]. The rheology of the ink determines the printability and quality of the final product [70]. An important consequence of high suspension viscosity is excessive heating of the printed material (there is a 30 °C difference between the center and the wall of the flow [71]).

Despite the advantages of nanothermite systems, safety concerns hinder the production and use of nano-energetic materials to a certain extent.

## 4. Trends and Directions for Further Research

The problems of additive manufacturing of HEMs can be divided into two groups: problems associated with the use of materials in the production process (ignition possibility, rheology, technical and economic parameters, etc.); the quality issues of the final products (mechanical properties, the need for post-processing, energy parameters compliance).

Table 1 summarizes the additive manufacturing technologies discussed in this review as well as their respective problems. It should be noted that the set of additive technologies is wider than we have considered. However, attempts to use them in the fabrication of items from reactive materials have not been observed in the literature.

Reactive material products from the additive manufacturing process have been proposed for a wide variety of applications. Different applications may require very different, sometimes oppositional, properties of reactive materials:

- Providing a high reaction rate (for example, a multichannel igniter [72]);
- Providing a low controlled reaction rate and the absence of gas formation [73];
- Releasing heat during the reaction (for example, destruction of microcircuits [74]);
- Generating gases (for example, micromotors and actuators [75]).

The design of processes for the additive manufacturing of products from high-energy nanocomposites is based on the requirements of a specific engineering task and is an example of engineering art. Here, it is necessary to:

- Choose an adequate 3D printing method;
- Investigate the feasibility of the processes pertinent to the preliminary preparation of materials, printing and post-processing while ensuring their compliance with safety requirements;
- Achieve the required mechanical and energy properties of the final product.

The research methods may include mathematical modeling or physical modeling, including the use of simulators of explosives.

Mathematical modeling of 3D printing processes is complex and multi-faceted. It must take into account many problems pertinent to mechanical engineering, heat and mass transfer, as well as chemical kinetics. Currently, there are many works devoted to the mathematical modeling of various additive manufacturing processes. The authors of [76] propose a mathematical model of heat transfer in Selective Laser Melting (SLM) [76]. In [77], a two-dimensional mathematical model of laser cladding with injection of Direct Metal Deposition (DMD) droplets has been developed. In [78], the temperature profile of the powder layer in the SLM process was calculated by the finite element method in a three-dimensional formulation. The research work [79] is also devoted to the mathematical modeling of the SLM process (for metal powders). The improved model takes into account laser power, scan speed, thermal conductivity and heat capacity of the powder. The authors of [80] consider the DIW process of orthopedic material printing using a relatively simple mathematical model in order to optimize printer parameters. Statistical analysis methods also find their use here. Thus, in [81], linear regression methods were used to assess the roughness, as well as the correlations between material filling and printing speed and correlations between layer height and temperature in the DIW process of ceramic materials. Research work [82] optimizes important parameters of the FDM process (layer thickness, assembly orientation, fill density and contour count) to improve the dimensional accuracy using hybrid statistical tools. Thus, mathematical modeling of the 3D printing processes is evolving, allowing for the optimization of these processes. However, there is a lack of research that would take into account the specifics of reactive materials in mathematical models of additive manufacturing. Still, the safety of the printing process associated with the possibility of explosive initiation remains an important issue.

The advantage of additive manufacturing of high-energy products lies not only in the ability to control the properties of products by changing the physicochemical parameters of the feedstock (for example, powder particles size). The ability to control geometric 3D shapes on a micro-scale is also valuable since the rate of combustion and detonation depends on them [83]. This opens new perspectives but also complicates the engineering task.

Thus, it can be assumed that the most important challenges in obtaining high-energy nanocomposites adapted for 3D printing technologies are: (i) materials safety and (ii) the viscosity of the binder-nanopowder system. Here, high viscosity is a result of the agglomeration of nanoparticles and their high specific surface area. One of the approaches to solving these problems lies in the deagglomeration and encapsulation of aluminum nanoparticles.

## 5. Promising Methods for Improving the Safety and Manufacturability of Nanopowders for 3D Technologies of High-Energy Materials

Increased reactivity of aluminum nanopowder is known to be one of the key characteristics that contribute to its extensive research and application in the composition of energy systems [84,85,86,87,88]. However, there are drawbacks limiting their application in additive manufacturing. One of these disadvantages is the increased sensitivity to the effects of oxidizing and corrosive environments due to the large specific surface area of the nanomaterials [89,90]. This disadvantage leads to a significant decrease in the content of active aluminum, thus lowering its energy characteristics in the energy system compositions.

Another disadvantage is the increased tendency of nanoparticles to agglomerate. This significantly reduces the viscosity of the powder-polymer system and complicates the preparation of materials for 3D printing. There are different approaches to solving this problem. For example, in Ref. [91], it was proposed to use copper oxide particles in the form of a wire mixed with nanoaluminum particles to reduce the size of agglomerates and increase the combustion rate of the mixture.

To solve this problem, we propose a new technology. It aims to modify the surface of aluminum nanopowder by creating a protective coating on it [92,93]. The protective layer will postpone direct contact between nanopowder particles till the moment of their active oxidation. It is obvious that the modifier should be applied to deagglomerated particles. Partial destruction of agglomerates can be achieved by homogenization under the effect of the flow of a liquid organic solvent medium containing surface modifier molecules. In the process of agglomerate disintegration, the interaction of modifier molecules and the surface of nanoparticles occurs, which results in the formation of a protective layer. It should be noted that even a partial replacement of micron-sized powders with aluminum nanopowder results in the reduction of mixture viscosity.

**Figure 8:**
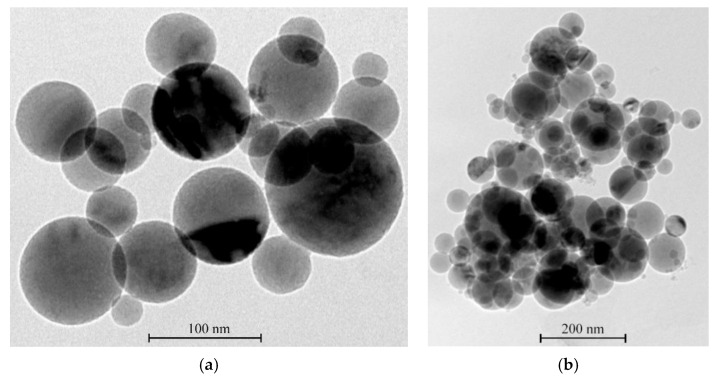
TEM image of aluminum nanoparticle size with magnification (**a**) ×60 × 10^4^ times and (**b**) ×30 × 10^4^ times.

For experimental research, we used aluminum nanopowders from Alex [94]. The aluminum nanoparticle size distribution histogram has a right-sided asymmetry and is close to the lognormal distribution (Figure 8a) [95]. The content of active aluminum in nanopowders is about 90% of the mass, and the specific surface of the nanopowder is S = 28.0 m^2^/g. Nanoparticles form micron-sized agglomerates (Figure 8b). There is a 2.10 nm thick layer on the surface of nanoparticles. This layer includes aluminum hydroxides: bayerite—α-Al (OH)_3_ and boehmite—γ-AlOOH.

To avoid the agglomeration of nanoparticles, surface modification is required. Thin layers (films) should be created on nanoparticle surfaces to prevent contact between them until the moment of their active oxidation. To apply such films to nanoparticle surfaces, agglomerates of nanoparticles must first be disintegrated.

The disintegration of nanoparticle agglomerates occurs during the intensive processing of powders exposed to the effect of the liquid organic solvent flow containing chemical surface modifiers. The additional agglomerate disintegration effect is caused by the interaction of modifier molecules with the surface of nanoparticles resulting in the formation of a film on the nanoparticle surfaces.

The substances presented in Table 2 have been selected as active modifiers.

This research has shown that in samples of nanopowders treated with 8-hydroxyquinoline, the content of the fine fraction relative to the initial aluminum nanopowder slightly increases. The share of nanoparticles less than 100 nm in size is 23% of their total number.

In the samples of nanopowders modified with pyrocatechol, the proportion of nanoparticles with a size less than 100 nm is 32% of their total number, while the difference between the fractions becomes less pronounced. Somewhat better disaggregation of nanopowders is achieved when using stearic acid. The fraction of particles less than 100 nm is 36% of their total number.

**Figure 9:**
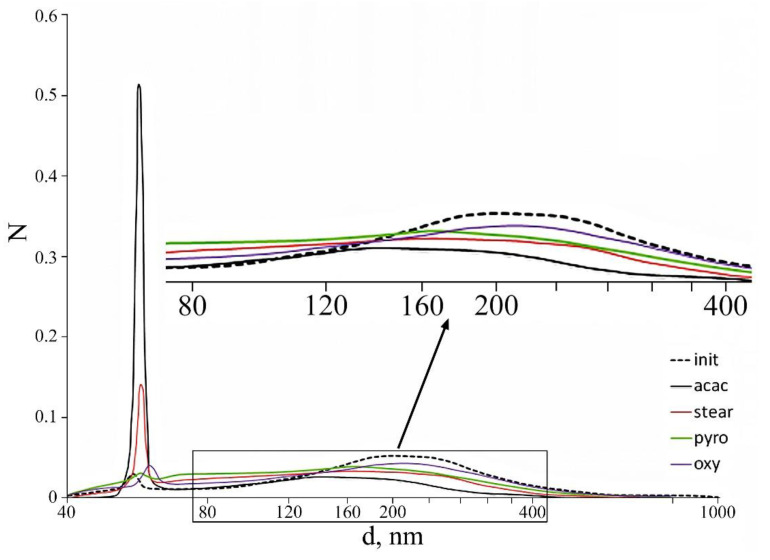
Size distribution of nanoparticle agglomerates.

The best dispersion is achieved when using acetylacetone, whereby the fraction of particles less than 100 nm is 56%. The data obtained show that the deagglomeration effect is determined by the bond strength between the modifier and the particle surface, as well as the difference in the intermolecular interaction of the modifiers used. Stearic acid forms unstable covalent compounds with surface aluminum cations, so its efficiency as a surface modifier is low. Pyrocatechol and 8-hydroxyquinoline readily polarize, which enhances the interaction of protective layers formed by these modifiers on the surface of nanoparticles with each other. Acetylacetone forms strong covalent bonds with superficial aluminum cations. Furthermore, acetylacetone molecules are characterized by low polarizability and strong intermolecular repulsion. Figure 9 shows particle and agglomerate size distributions in the aluminum powders after their treatment with different modifiers.

A protective layer of acetylacetone on the surface of nanoparticles protects (shields) the surface of nanoparticles, thereby reducing the level of interactions between them. Thus, it was shown that acetylacetone has the highest efficiency as a surface modifier. This ensues from the data obtained that the creation of protective layers (films) that prevent aluminum nanoparticle agglomeration requires surface modifiers in order to form bonds with superficial metal atoms. These modifiers make it possible to reduce the surface energy of the dispersed phase.

For microencapsulation, the best methods are colloidal methods of material fabrication. Those rely on the deagglomeration of nanoparticles in a liquid medium in the presence of a polymer, sorption of the polymer on nanoparticle surfaces followed by solvent removal. These methods are simple and technologically mature, while allowing for the achievement of almost complete deagglomeration and, therefore, uniform coating of nanoparticles with a polymer layer.

In this work, the following components were used as polymers for surface modification:

1. HTPB—hydroxyl-terminated polybutadiene with low molecular weight.
2. MPVT is a copolymer of 1-methyl-5-vinyltetrazole, 2-methyl-5-vinyltetrazole, *N*-allyl-5-vinyltetrazole and acrylonitrile.

Petroleum ether (70/100) was used as a solvent for the HTPB polymer, and dimethylformamide and ethyl acetate (butyl acetate) were used for the MPVT polymer.

It has been experimentally established that the highest efficiency of the microencapsulation process is achieved in units with periodic interruptions in the liquid medium flow. Periodic liquid medium flow interruptions lead to multiple discontinuities in the liquid medium flow (the effect of acoustic cavitation). Accordingly, ultrasonic vibrations are generated in a wide frequency range, which is very effective in the case of nanoparticles. Under the effect of the generated liquid medium flows, the aggregates of nanoparticles are disintegrated, and the mass transfer of reagents is intensified.

Microencapsulation of nanoparticles was performed by the following technique:

Solvent (300 mL), 20.00 g of powder and 100 mg of polymer were placed in a round-bottom flask with a capacity of 500 mL. The mixture was processed in an HG-15D high-speed homogenizer over 30 min at a stirring speed of 5000 rpm. The solvent was removed on an IKA 10 RV rotary evaporator, and the resulting powder material was dried at a pressure of 1 Torr over 16 h.

**Figure 10:**
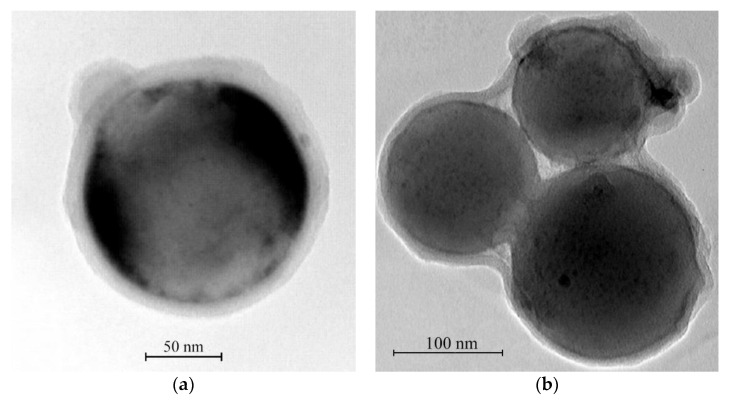
TEM images of modified aluminum nanopowder with magnification (**a**) ×12 × 10^5^ times (**b**) and ×60 × 10^4^ times.

Figure 10 shows a general view of surface-modified aluminum nanopowders. The figure shows that aluminum nanopowder particles are covered with a uniform polymer film. Such a film remains stable until the onset of the active phase of aluminum oxidation. This facilitates the solution of material safety problems in the technological processes of 3D printing of high-energy systems. In addition, the created film reduces the specific surface area of nanopowders. This allows for obtaining pastes with a lower viscosity as well as implementing 3D printing in a more streamlined and efficient manner.

## 6. Conclusions

- Additive manufacturing technologies used with high-energy materials have been considered based on the analysis of literary sources.
- The advantages and disadvantages of these technologies from the point of view of using high-energy materials for printing have been shown.
- Requirements for the preparation of materials for printing by using each of the technologies under consideration have been formulated.
- It has been shown that the chemical reactivity of HEMs significantly complicates the problem of additive manufacturing, development and optimization of 3D printing methods. In addition to customary complications in the development of these methods (for example, high suspensions viscosity), there are specific problems associated with the nature of the reactive substances. First and foremost, it is the possibility of initiating reactive materials in the 3D printing process.
- This paper proposes a method for the microencapsulation of nanosized aluminum powders with polymeric materials of different chemical compositions. It has been shown that polymers form a continuous homogeneous layer (film) on the surface of nanoparticles. The research has demonstrated an almost complete degree of deagglomeration of microencapsulated aluminum powders. Such powders open the potential for creating new systems for safe 3D printing using high-energy materials.
- The development of new paste formulations for the 3D printing of HEMs is the subject of further research, which will be published in a separate paper.

## Figures and Tables

**Table 1:** Additive manufacturing technologies for HEMs and problems pertinent to their use.

AM Technology	Advantages	Problems and Material Requirements	Sources
DIW	Relatively low process temperatures Relative simplicity High resolution (up to 1 μm)	Viscosity of highly filled inks Uniform distribution of particles in the ink Suspension stability Possibility of ignition and detonation during fabrication	[21,22,23,24,25,26,27,28,29,70,71]
FDM	High viscosity of the feedstock Inexpensive commercial printers	Unsatisfactory mechanical properties of the products, low resolution Balance of material viscosity and its reactivity Possibility of ignition and detonation due to the relatively high temperature of the filament	[30,31,32,33,34,35,36,37,38,39]
Photopolymerization methods (SLA/DPL)	High resolution and high performance No toxic hardeners used	The need for polymers curable by UV or visible light Low viscosity of materials	[30,40,41,42,43,44,45,46,47,48]
Binder jetting	No supporting structures required Ability to use leftover material for a new print process Low process temperatures	Post-processing required to eliminate mechanical defects and increase strength Removal of excess reactive powder Product fragility	[14,49]
SLS	High precision manufacturing of products of complex geometric shapes High speed and performance Excellent mechanical properties of products No wastes	High equipment costs Intense exposure to IR laser may initiate explosives	[50,51]

**Table 2:** Content of modifiers.

Coating	The Amount of Organic Substances, wt.%
substances, wt.	0.5
pyrocatechol	0.5
8-oxyquinoline	0.5
stearic acid	3.0

## Data Availability

The data presented in this study are available on request from the corresponding author.
